# Study Destination Choice and Career Preferences of International Students at China Pharmaceutical University

**DOI:** 10.3390/pharmacy10060149

**Published:** 2022-11-10

**Authors:** Raphael N. Alolga, Said Abasse Kassim, Pierre Dramou

**Affiliations:** 1 State Key Laboratory of Natural Medicines, School of Traditional Chinese Pharmacy, Department of Pharmcognosy, China Pharmaceutical University, Nanjing 210009, China; 2 Département de management, Faculté des sciences de l’administration, Université Laval, 2325 Rue de l’Université, Québec, QC G1V 0A6, Canada; 3 Department of Analytical Chemistry, School of Science, China Pharmaceutical University, Nanjing 210009, China

**Keywords:** career preference, study destination, China, pull factors, international students, pharmacy

## Abstract

This study had two main thematic aims: first, to determine the factors that influenced the choice of international students at China Pharmaceutical University (CPU) to consider China as a study destination; second, to determine the career preferences of international students upon completion of their various programs of study. As a cross-sectional study, relevant data were collected from undergraduate pharmacy students and postgraduates using a self-administered questionnaire. On the whole, the five most important pull factors that influenced the choice of China as the study destination for the respondents were: (1) quality of education, (2) quality of academic staff, (3) security, (4) desire to obtain a certificate from a foreign country and (5) availability of scholarship opportunities. With respect to the career choices, the top three career preferences of the international students were: (1) to work in the pharmaceutical industry (i.e., pharmaceutical manufacturing companies), (2) to practice clinical pharmacy and (3) to seek employment opportunities outside their countries. This study provides additional details on why China has gradually become a study destination of choice for international students. The career preferences of students could be useful in the design of academic programs that could meet their job aspirations.

## 1. Introduction

The People’s Republic of China (China) has gradually emerged as a study destination of choice for international students [1,2]. This increased trend of educational preference by foreigners seeking higher education is the result of the continual institution of pragmatic, meticulous and convivial educational policies by the leadership of the country [1,2]. Congruent with the Reform and Open-Up Policy of 1978, China has undergone rapid and effective transformational changes in various key areas (economic, educational, social sectors, etc.) [2,3]. An open door has been extended to foreigners to study various programs by the provision of scholarships and other incentives [2]. The recruitment of students is not so much to generate revenue for the country as it is to educate and inculcate in the students the values, philosophy and cultural norms of the Chinese people. This essentially would lead to a holistic education that produces graduates that are knowledgeable about China, friendly to and fond of China [4]. The introduction of English-taught programs by many universities as well as the global rankings of Chinese universities partly account for the surge in the number of international students in China [5]. The aforementioned (i.e., good economic conditions, availability of scholarships, global recognition of Chinese universities) form part of various factors that determine the choice of destination for foreign education, known as the “push-pull” factors [6,7]. The “push” factors pertain to conditions within the home countries of the students that tend to push them to seek education in other countries. The “pull” factors relate to various favorable and enticing conditions in the foreign countries of choice that attract and pull the students there [3,6,7] Though the ideal “pull-push” model generally throws light on the external factors that influence students’ mobility, the focus is usually at the macro level with little to no consideration on equally important issues at the micro level (i.e., issues at the individual level) [8]. Factors pertaining to the individual student such as academic ability, personal motivation and aspiration, gender, age and socioeconomic status are as crucial as the external factors such as political instability, lack of educational and employment opportunities in the home countries of the students in their choice of study destinations [9,10,11]. To this end, some researchers have sought to incorporate various micro- and macro-level factors into the “pull-push” model (to produce a modified “pull-push” model) in an attempt to facilitate a better understanding of the factors that influence students’ mobility and choice of study destinations [12,13,14]. Various “pull-push” factors have been reported by different researchers in respect to the study destination choices of international students from different countries. Though these factors tend to differ in terms of the priorities of the students, they are usually not entirely new regardless of the study destination. The findings of studies conducted in countries such as Malaysia [15], the United Arab Emirates [16], the United States of America [7], China [2], Iran [17], India [18], Korea [19], Australia [20], etc., identified several notable factors, among which were the quality of education, international reputation (ranking) of the university, cost of living (and cost of education), availability of scholarships, ease of gaining admission, ease of obtaining visas and prospects of better employment opportunities during and after completion of program of study. With particular reference to China as a study destination, the few studies on students’ mobility were not focused on specific program(s) of study such as medicine or pharmacy [2,21,22]. Studies on medical or pharmacy education, were not focused on determining the “pull-push” factors that made China a study destination for international students [23,24]. To the best of our knowledge, there is no scientific literature on the probable factors that make China a preferred destination by international students for the study of a specialized medical program such as pharmacy.

China Pharmaceutical University (CPU) holds the enviable and iconic position as the citadel of pharmacy education in China. Founded in 1936, CPU ran China’s first four-year pharmacy program and higher degrees in pharmacy education. It is part of the list of “211 projects” of key universities and the “double first-class” universities in China. The university also enjoys global recognition in respect of its position on the various global academic rankings [25]. For instance, the pharmacology and toxicology disciplines of CPU ranked 1st in China, 34th globally and among the top 1% globally in 2020 on the Essential Science Indicators (ESI) ranking [25]. In the same year, these two disciplines also ranked 18th globally and 1st in Asia on the US New World University ranking [25]. CPU offers various pharmacy-related undergraduate and postgraduate programs, notable among which are the English-taught Bachelor of Pharmacy and Bachelor of Clinical Pharmacy programs as well as various Master of Science and PhD programs for which English is the medium of instruction (meant for international students) [25].

The pharmacy profession has evolved over the centuries and spread its tentacles from general compounding to patient-centered care delivery. Hence, aside from the traditional role as a medicine specialist, the pharmacist is now adequately furnished with the requisite knowledge to work in diverse fields within or related to the pharmaceutical sector. The role of the current pharmacist is succinctly captured by the “nine-star pharmacist” concept promulgated by the World Health Organization (WHO) as: caregiver, decision-maker, communicator, manager, leader, life-long learner, teacher, researcher and entrepreneur [26,27]. The “nine-star pharmacist”, however, evolved from the previous “seven-star pharmacist” concept upon the addition of “researcher” and “entrepreneur” as part of the roles of the modern pharmacist [26,27]. The trainee (student) pharmacist is thus adequately trained to work in various sectors of the health care delivery system. Pharmacy students could therefore be guided in their choice of career paths by counselors and educators, and they could direct them on how to achieve the same.

In this study, we sought to gain insights from international students at CPU on two thematic research areas: (1) the possible factors that influenced their choice of China as a study destination and (2) their career choices upon completion of their programs of study. Implicit in these two main areas would be answers to questions such as: (a) Why not choose any of the English-speaking Western countries as study destinations? (b) Is there a gender or age disparity in the choice of China as a study destination? (c) Is there a gender or age disparity in the career choices of the students? (d) Is there any link between the level of education (undergraduate or postgraduate) and the career choices of the students? Taken together, we aimed to fill possible gaps in the published literature on students’ career preferences and study destination choices with a particular emphasis on the pharmacy profession. To accomplish the first thematic aim of this study, we adopted the concept of the “modified pull-push” model in the design of our questionnaire while the content of the questionnaire addressing the second thematic aim was derived from previous credible studies with similar research aims [2,28].

## 2. Materials and Methods

### 2.1. Study Design and Setting

This was a descriptive cross-sectional study that was conducted between June and July 2022 at CPU, located in the capital of Jiangsu province, Nanjing, China.

### 2.2. Study Population

The study focused on international students at CPU studying various pharmacy-related programs at the undergraduate and postgraduate levels (masters and PhD).

### 2.3. Sample Size and Sampling

The sample size was calculated using the free online software, Raosoft, with 95% confidence interval, 5% margin of error and 50% expected response [29]. Based on data available to the authors, the accessible study population was about 452 students (in China and abroad), hence, a minimum sample size of 154 was required (undergraduate, 276; masters students, 136; PhD candidates, 40). Using the two probability sampling methods, the participants were thus selected (i.e., stratified and systematic sampling). The participants were mainly categorized into three strata according to their academic levels of study (i.e., undergraduates, masters and PhD) and sample size for each stratum obtained by systematic sampling (using Excel). The respondents were from various parts of the world, including Asia (12.3%), East Africa (17.5%), West Africa (27.9%), Southern Africa (30.5%), Europe (1.3%), etc.

### 2.4. Data Collection

Data were collected using a structured self-administered questionnaire that was developed with inspiration from previous studies with credible methodological approaches [2,28]. The questionnaire consisted of five main parts: A, B, C, D and E. The first part took an inventory of the sociodemographic characteristics of the students including gender, age, level of study and source of funding. Part B entailed a list of possible factors that influenced the choice of China as a study destination for pharmacy. This part consisted of 19 items to which the participants had to respond to based on a 3-point significance scale: 1, most important; 2, somewhat important; 3, not important. Part C of the questionnaire sought to find out from the participants their probable reason(s) for not choosing to study in a Western country. For that, 8 possible factors were to be ranked by the participants using the 3-point Likert scale mentioned earlier. The fourth part (part D) explored the career preferences of the students upon completion of their studies. From a total of 12 items, the students were required to select their top 3 options in order of importance as follows: 1, 1st choice; 2, 2nd choice; 3, 3rd choice. The final part (part E) of the questionnaire aimed to determine the probable factors that influenced the career choice of the students in part D. Under this part, the possible reasons were categorized under three main factors; faculty-related influences, personal-related influences and job-related influences. A total of 16 items were provided for inputs of the participants based on a 5-point Likert scale: 1, strongly agree; 2, agree; 3, neutral; 4, disagree; 5, strongly disagree (Supplementary Materials).

The validity of the questionnaire was assessed by two senior researchers and pretested by 22 selected students. Suggested changes therefrom were then effected in the final version of the questionnaire. The final version of the questionnaire was then uploaded onto an online platform using WeChat (a mobile phone App) and a QR code generated (Supplementary Materials). The QR code was then sent to the various participants to scan and complete the required information. The settings of the electronic questionnaire were preset so that each participant could submit only one completed questionnaire. Moreover, each part of the questionnaire had to be completed before proceeding to the next and one could only submit a completed questionnaire (i.e., incomplete information could not be submitted). Completed questionnaires by the participants were collected between 28 June 2022 and 15 July 2022.

### 2.5. Data Analysis

The collated data were analyzed using the Statistical Package for Social Sciences (SPSS). Statistically significant difference or association between independent (i.e., gender, age, etc.) and dependent (i.e., career choice, study destination, China) variables was assessed using the chi-square test and a *p* value < 0.05 was considered statistically significant.

## 3. Results

### 3.1. Sociodemographic Characteristics of Participants

As summarized in Table 1, of the 154 students that participated in this study, 74 (48.1%) were female while 80 (51.9%) were male. The age range of the participants were ≤20 years (13.6%), 21–30 years (76.0%) and >30 years (10.4%).

With regard to the level of study, the majority of the respondents were undergraduates, 109 (70.8%), while 29 (18.8%) and 16 (10.4%) were pursuing master’s and doctoral degrees, respectively. The respondents were from various parts of the world, including Asia (12.3%), East Africa (17.5%), West Africa (27.9%), Southern Africa (30.5%), Europe (1.3%), etc. Finally, 77.9% of the respondents were on various scholarship schemes while 22.1% were self-funded students.

### 3.2. Factors Influencing Choice of China as Study Destination

The responses of the participants clearly reflected the factors that were important to them in their choice of China as a study destination. The most important “pull” factor to the students in their choice of a study destination was the quality of the learning environment (Table 2). Most of the respondents (79.9%) considered the quality of the university as the most important factor that influenced their choice of a study destination. Closely related to that was the quality of the academic staff of the university. This factor ranked second on the list by 77.92% of the students. The third most important factor that influenced the choice of study destination was the security of the potential study destination. The desire to obtain an academic qualification from a foreign country and the availability of scholarships ranked fourth and fifth on the significance scale, respectively. The other factors that influenced the choice of China as a study destination are appropriately ranked in Table 2.

With regard to the probable factors that excluded Western countries as study destinations, the high cost of education, high cost of living, difficulty in gaining admission, difficulty in obtaining student visas and insecurity ranked first, second, third, fourth and fifth, respectively, on the scale of significance (Table 2).

### 3.3. Gender Disparities in Factors Influencing Choice of Study Destination

We sought to determine any possible gender disparities in the reasons that accounted for the choice of study destination earlier outlined (Section 3.2). Table 3 provides a comprehensive summary of the gender distribution for all options of the various factors that influenced the choice of study destination. Though the percentage of male and female respondents for the various factors differed, the differences were largely not significant (statistically). For instance, 52.8% of male and 47.2% of female respondents selected the option “most important” for the first ranked reason that influenced their choice of China as a study destination (i.e., the quality of university learning environment). For the second ranked reason (quality of academic staff), 54.2% of male and 45.8% of female respondents chose the option of “most important” reason that influenced their choice of study destination. Similarly, for the first ranked reason for the respondents not studying in a Western university, 51.1% of male and 48.9% of female respondents selected the option “most important”.

### 3.4. Career Choices of Respondents and Influence of Gender Differences

As part of the aims of this study, we sought to determine the career choices of the students after graduation (Table 4). To this end, they were required to select from a pool of career options their top three career preferences (ranked first to third choice). On the whole, the first choice of career preference by most of the respondents was to work in the pharmaceutical industry (i.e., pharmaceutical manufacturing companies). Their second choice was to practice clinical pharmacy (i.e., patient care in hospitals, clinics etc.). However, if for some reason they were not able to secure jobs in these areas, they would prefer to seek job opportunities outside their home countries (i.e., their third choice).

We also aimed to determine if there were gender disparities in the career choices of the respondents. In this regard, for the majority of the male respondents, their first choice of career preference was to work in academia and research, while the practice of clinical pharmacy was their second choice. In the instance whereby they do not gain employment in these two areas, they would seek employment outside their countries of origin. The first career choice for the majority of the female respondents was clinical pharmacy practice while work in the pharmaceutical industry was their second choice on the scale of career preference. Similar to their male counterparts, the majority of the female respondents also indicated their willingness to seek employment opportunities in foreign countries.

### 3.5. Influence of Age of Respondents on Career Choices

Since the respondents were of different ages, we sought to determine if the age of the respondents played a role in their future career choices (Table 5). We grouped the participants into the following age groups; ≤20 years, 21–30 years and >30 years. Hence, for the first choice of career preference by age group, respondents of ≤20 years, 21–30 years and >30 years, respectively, chose potential careers in the pharmaceutical industry and academia and research. For the second choice of future career preferences, the majority of the respondents in the various age groups chose to work as clinical pharmacists. For the third career choice, the respondents of ≤20 and 21–30 years indicated their intentions to seek employment opportunities outside their countries of origin if they are unable to gain employment in their first and second choice of career options. The respondents of age >30 years were equally divided on the three career options (for the third choice): work in the drug regulatory bodies, pharmaceutical industry or seek employment outside their countries of origin.

### 3.6. Influence of Academic Level of Study of Respondents on Career Choices

The respondents were pursuing various academic programs at the undergraduate, master’s and PhD levels. We therefore sought to determine the possible impact of the academic level on the career choices of the respondents (Table 6). For the first choice of career preferences, the majority of the undergraduate students hope to work in various capacities in the pharmaceutical industry while the master’s students and PhD candidates aim to work in academia and research upon graduation. Most of the undergraduate and master’s students hope to work as clinical pharmacists if they do not get employment opportunities in the pharmaceutical industry (second choice). The PhD candidates hope to work in the pharmaceutical industry in the event that they are not employed in academia and research. In the event that the undergraduate respondents do not get opportunities in their first and second choices, they would seek employment outside their countries of origin. The third choice of career preference by the master’s students and PhD candidates were to work in the pharmaceutical industry and drug regulatory bodies, respectively.

## 4. Discussion

We report for the first time (to the best of our best knowledge), the factors that informed the decisions of international students in their selection of China (and not a Western country) as the study destination for pharmacy and pharmacy-related programs. Moreover, this study reports for the first time, the career preferences of international students studying pharmacy and various pharmacy-related programs at China Pharmaceutical University. These findings therefore add to the body of available literature on factors that influence students’ mobility and career preferences. Using the study of pharmacy and pharmacy-related programs as a case study, this study essentially provides additional details on why China has gradually emerged as a destination of choice for international students. Our findings could be useful for institutional curriculum design of academic programs that would offer quality training and better prepare students to meet their career aspirations in the pharmaceutical sciences.

Until recently, English-speaking Western countries such as the UK, the USA, Australia and Canada were the first-choice study destinations for international students from various countries including China. China has gradually become a destination of choice for many students [1,2]. This transformation from being the biggest source of international students to becoming an attractive study destination has not been by mere serendipity but the culmination of consistent governmental efforts that have been rolled out over the years [1,2].

A wide array of factors have been reported to have influenced the choice of Western countries as the study destinations for many international students including the quality of education, recognition of qualifications, ease of admission, employment opportunities during and after study, safety of the learning environment, etc. [2]. As part of the broader aims of this work, we sought to determine the possible factors that influenced the choice of China as a study destination for international students at China Pharmaceutical University, Nanjing. In this study, the five most important factors (“pull” factors) that influenced their choice of China as a study destination were: (1) quality of education, (2) quality of academic staff, (3) security, (4) desire to obtain a certificate from a foreign country and (5) availability of scholarship opportunities. These “pull” factors have been reported among others in previous studies on international students seeking higher education in English-speaking Western countries [6,30,31,32,33]. For instance, Ahmad and Hussain (2017) who investigated the possible reasons for the mobility of international students to the Middle East (United Arab Emirates) found the following as the most important “pull” factors: quality of the learning environment, cost issues (living and studying), institutional reputation and opportunity for personal development [16]. Wadhwa R who sought the views of Indian students on the possible “pull” factors to seeking foreign education reported the following: a better standard of living (in destination country), better education abroad, high prospects of employment and income and prestige of a foreign degree [18]. According to Eder et al. (2010), the “pull” factors for international students at Southern University, USA, were succinctly captured as college issues, physical geography and the US culture [7]. Finally, Oguche D (2022) who sought the opinions of Nigerians studying in the UK on the underlying reasons and motivations for their choice of study destination found that foreign education offered the students a competitive advantage, international exposure and better education among other merits [34]. Contrary to previous studies, we sought to determine if the “pull” factors were influenced by the gender of the respondents to any considerable extent. We had a fairly even representation of male (51.9%) and female (48.1%) respondents. There were generally no statistically significant differences in respect of the gender of the respondents. Hence, gender differences of the respondents to a large extent did not influence the factors they considered as the most important “pull” factors that finally culminated in their study destination choices. Similar to the study by Ahmad and Shah (2018), we examined the possible reasons that could have accounted for the international students not considering to study in a Western country. The five most important factors that excluded the Western countries as study destinations were the high cost of education, high cost of living, difficulty in gaining admission, difficulty in obtaining student visas and insecurity. Our findings, which are almost a replica of that of Ahmad and Shah (2018), lend credence to the significance of their findings in terms of the factors that influence the study destinations of international students [2]. They reported the following as the five most important factors that informed the decisions of their participants not to consider studying in a Western country: high cost of degrees, high cost of living, difficulty in obtaining admissions, difficulty in getting student visa and concerns about safety and wellbeing [2]. A perusal of the respondents’ countries of origin revealed that the majority of them were from Africa. Hence, the availability of scholarship opportunities in China juxtaposed with the high cost of education and difficulties in obtaining admission (and visas) from English-speaking Western countries [7] make China a more economically attractive study destination to African students and their guardians.

To address the second thematic aim of this study, we got the responses of the participants on their career preferences upon completion of their various programs. Holistically, their top two career choices were to work in the pharmaceutical industry (i.e., pharmaceutical manufacturing companies) and practice clinical pharmacy. However, in the event that they did not secure jobs in any of these two fields, the respondents indicated their willingness to seek employment opportunities in pharmacy-related fields outside their countries of origin. With respect to gender-specific preferences, the first and second choices of the majority of the male respondents were, respectively, to work in academia and research and practice clinical pharmacy. For the third choice, the male respondents indicated that they would seek employment outside their home countries in case they did not get their first and second career choices. For the female respondents, clinical pharmacy practice and work in the pharmaceutical industries were their first and second career choices. For them as well, in case they did not get their first and second choices, the female respondents would also seek employment outside their countries of origin. These findings are quite unique with some similarities and differences to previous studies [35,36,37,38]. For instance, Arhab et al. (2022) who recruited Sudanese undergraduate pharmacy students with a very high female-to-male ratio (86%:14%), reported clinical pharmacy practice, academia and research and work in the pharmaceutical industry as their first, second and third career preferences, respectively [28]. Their findings differ from this current study when the entire study population (male and female respondents together) are compared with theirs. However, the first choice of their respondents is the same as that for the female respondents of this study, i.e., clinical pharmacy practice. Moreover, the third choice of career preference for their respondents is the second choice of the female respondents of this study. The high female-to-male ratio of their study sample could have accounted for this similarity. Another possible reason could be the fact that their study participants were only undergraduate pharmacy students. In this study, we also examined the career preferences of the respondents on the basis of their academic levels of study. Hence, we obtained the responses of undergraduates, master’s students and PhD candidates in respect of their top three career choices upon graduation. As earlier indicated, the top three career preferences of the undergraduate cohort of this study were, respectively, to work in the pharmaceutical industry, practice clinical (hospital) pharmacy and seek job opportunities outside their home countries if they do not gain employment in their first two choices. This finding differs from that of Arhab et al. (2022) and others such as Hasan et al. (2010), Alhomoud et al. (2019) and Beedemariam et al. (2014) who conducted similar studies in Malaysia [36], Saudi Arabia [37] and Ethiopia [38]. The first choice for the postgraduate students (master’s and PhD) was to seek employment in the area of academia and research. This preference is quite understandable in the sense that postgraduate education in pharmacy adequately prepares one for work in any academic or research institution. Another interesting observation made between the career preferences of the undergraduates and postgraduates relates to the average ages of the students. The postgraduates were generally older (21–30; >30 years) than the undergraduates (≤20). The age group of 21–30 years preferred to work in the pharmaceutical industry (first choice) or practice clinical pharmacy (second choice). However, they were willing to seek employment outside their countries of origin if they did not get their first and second career choices. However, for the >30 years age group, most of whom were PhD candidates, their preferred career choices were academia and research (first choice) and work in the pharmaceutical industry (second choice). For the third career choice, they were generally equally divided between three options, to work in drug regulatory bodies, pharmaceutical industries or to seek employment outside their home countries. The differences in the career preferences between the undergraduates and postgraduates could be due to possible experiential differences. The expectations of the postgraduates, particularly the PhD candidates, most of whom would have had some level of prior work experience, were more focused and realistic. The general intentions of most people who enroll in pharmacy-related PhD programs are mainly to work in research institutions (or research and development at pharmaceutical companies) or academia. Similarly, the master’s students, some of whom might have had a stint in the work environment, tend to study specific programs that better equip them for specific roles in their current workplaces or for future job opportunities. However, the undergraduate who is yet to have any work experience might change jobs a couple of times after graduation before finally settling on a long-term career path.

## 5. Conclusions

In conclusion, the five most important pull factors that influenced the international students’ choice of China as a study destination were the quality of education, quality of academic staff, sense of security (safety) at the study destination, desire to obtain a certificate from a foreign country and availability of scholarship opportunities. The five most important factors that excluded Western countries as study destinations were the high cost of education, high cost of living, difficulty in gaining admission, difficulty in obtaining student visas and perception of insecurity at the study destination. Though the choice of China as a study destination differed with the age and gender of the respondents, the influence of these two factors was not statistically significant. The top three career preferences of the international students were to work in the pharmaceutical industry (i.e., pharmaceutical manufacturing companies), practice clinical pharmacy and seek employment opportunities outside their countries of origin in the event that they were not able to gain employment in the first two career options. Though there were differences in the career choices of the respondents on the basis of gender, age and educational level, the differences were largely not statistically significant.

The main limitation of this study stems from the fact that it was conducted in only one university (CPU); hence, our findings cannot be generalized for all international students in China. In future studies, it would be worthwhile covering other universities in China where pharmacy and pharmacy-related programs are run. Furthermore, since our study design was a cross-sectional descriptive study, causality could not be directly inferred.

## Figures and Tables

**Table 1:** Sociodemographic characteristics of respondents.

Variable	Frequency		*p*-Value
	N (154)	(%)	
**Gender**			0.534
Female	74	48.1	
Male	80	51.9	
**Age (year)**			0.112
≤20	21	13.6	
21–30	117	76.0	
>30	16	10.4	
**Level of Study**			0.822
Undergraduate	109	70.8	
Master	29	18.8	
Doctorate	16	10.4	
**Place of Origin**			0.466
Asia	19	12.3	
East Africa	27	17.5	
West Africa	43	27.9	
North Africa	15	9.7	
Southern Africa	47	30.5	
Europe	2	1.3	
South America	1	0.6	
**Source of Funding**			0.814
Self-funded	34	22.1	
Scholarship	120	77.9	

**Table 2:** Factors influencing choice of study in China and not in a Western country.

	Mean	Rank	Most Important (%)	Somewhat Important (%)	Not Important (%)	*p*-Value
Factors influencing choice to study in China						
The quality of university learning environment	1.2078	1	123 (79.9)	30 (19.5)	1 (0.6)	0.782
The quality of academic staff	1.2273	2	120 (77.92)	33 (21.43)	1 (0.65)	0.395
Full or partial scholarship	1.3377	5	109 (70.78)	38 (24.68)	7 (4.55)	0.424
Wanting to obtain international qualifications	1.2468	4	119 (77.27)	32 (20.78)	3 (1.95)	0.408
Being able to learn Mandarin language	1.9481	17	46 (29.87)	70 (45.45)	38 (24.68)	0.199
Cost of living (e.g., accommodation, food)	1.4481	7	95 (61.69)	49 (31.82)	10 (6.49)	0.009
Personal safety and wellbeing	1.2338	3	123 (79.87)	26 (16.88)	5 (3.25)	0.392
Ranking of the university	1.3766	6	101 (65.58)	48 (31.17)	5 (3.25)	0.156
The growth of Chinese economy	1.6623	13	68 (44.16)	70 (45.45)	16 (10.39)	0.490
Learning Asian culture	1.9156	16	45 (29.22)	77 (50.00)	32 (20.78)	0.330
Trade agreements between my country and China	1.7403	15	67 (43.51)	60 (38.96)	27 (17.53)	0.347
Employment prospects in Asia or beyond	1.5974	11	80 (51.95)	56 (36.36)	18 (11.69)	0.372
Ease of entry	1.5065	9	92 (59.74)	46 (29.87)	16 (10.39)	0.514
Easy to get study visa	1.4610	8	97 (62.99)	43 (27.92)	14 (9.09)	0.841
Being able to start business with Chinese counterpart	1.6688	14	77 (50.00)	51 (33.12)	26 (16.88)	0.495
Low cost of the degree	1.6169	12	76 (49.35)	61 (39.61)	17 (11.04)	0.836
Incentives for international students to study in China	1.5519	10	80 (51.95)	63 (40.91)	11 (7.14)	0.404
My parents want me to study in China	2.1169	18	40 (25.97)	56 (36.36)	58 (37.66)	0.254
Some of my friends are studying in China	2.1623	19	42 (27.27)	45 (29.22)	67 (43.51)	0.339
Reasons for not considering to study in Western university						
Cost of education is high	1.5390	1	92 (59.74)	41 (26.62)	21 (13.64)	0.938
Cost of living is high	1.5390	2	90 (58.44)	45 (29.22)	19 (12.34)	0.882
Difficult to get admissions in Western universities	1.7857	3	65 (42.21)	57 (37.01)	32 (20.78)	0.608
Hard to get student visa	1.8506	4	58 (37.66)	61 (39.61)	35 (22.73)	0.194
Worried about my safety and wellbeing	1.8961	5	58 (37.66)	54 (35.06)	42 (27.27)	0.827
Far from my home country	2.4740	7	24 (15.58)	33 (21.43)	97 (62.99)	0.652
I may not integrate with Western cultures	2.5065	8	18 (11.69)	40 (25.97)	96 (62.34)	0.680
I may feel isolated	2.4481	6	28 (18.18)	29 (18.83)	97 (62.99)	0.495

**Table 3:** Gender disparities in the factors influencing choice of study destination.

	Rank	Most Important (%)		*p*-Value	Somewhat Important (%)		*p*-Value	Not Important (%)		*p*-Value
Factors influencing choice to study in China		Male	Female		Male	Female		Male	Female	
The quality of university learning environment	1	65 (52.8)	58 (47.2)	0.323	15 (50.0)	15 (50.0)	0.595	0 (0.0)	1 (100.0)	-
The quality of academic staff	2	65 (54.2)	55 (45.8)	0.714	15 (45.5)	18 (54.5)	0.656	0 (0.0)	1 (100.0)	-
Full or partial scholarship	5	61 (56.0)	48 (44.0)	0.267	17 (44.7)	21 (55.3)	0.601	2 (28.6)	5 (71.4)	0.19
Wanting to obtain international qualification	4	62 (52.1)	57 (47.9)	0.398	18 (56.3)	14 (43.8)	0.536	0	3 (100.0)	-
Being able to learn Mandarin language	17	25 (54.3)	21 (45.7)	0.515	32 (45.7)	38 (54.3)	0.263	23 (60.5)	15 (39.5)	0.162
Cost of living (e.g., accommodation, food)	7	46 (48.4)	49 (51.6)	0.935	31 (63.3)	18 (36.7)	0.330	3 (30.0)	7 (70.0)	0.833
Personal safety and wellbeing	3	63 (51.2)	60 (48.8)	0.564	14 (53.8)	12 (46.2)	0.494	3 (60.0)	2 (40.0)	0.800
Ranking of the university	6	55 (54.5)	46 (45.5)	0.576	22 (45.8)	26 (54.2)	0.469	3 (60.0)	2 (40.0)	0.800
The growth of Chinese economy	13	42 (61.8)	26 (38.2)	0.283	31 (44.3)	39 (55.7)	0.482	7 (43.8)	9 (56.3)	0.351
Learning Asian culture	16	30 (66.7)	15 (33.3)	0.427	33 (42.9)	44 (57.1)	0.289	17 (53.1)	15 (46.9)	0.710
Trade agreements between my country and China	15	40 (59.7)	27 (40.3)	0.769	26 (43.3)	34 (56.7)	0.325	14 (51.9)	13 (48.1)	0.259
Employment prospects in Asia or beyond	11	41 (51.2)	39 (48.8)	0.363	26 (46.4)	30 (53.6)	0.037	13 (72.2)	5 (27.8)	1.00
Ease of entry	9	45 (48.9)	47 (51.1)	0.587	25 (54.3)	21 (45.7)	0.921	10 (62.5)	6 (37.5)	0.313
Easy to get study visa	8	48 (49.5)	49 (50.5)	0.868	23 (53.5)	20 (46.5)	0.543	9 (64.3)	5 (35.7)	0.438
Being able to start business with Chinese counterpart	14	40 (51.9)	37 (48.1)	0.760	22 (43.1)	29 (56.9)	0.506	18 (69.2)	8 (30.8)	0.196
Low cost of the degree	12	39 (51.3)	37 (48.7)	0.913	32 (52.8)	29 (47.5)	0.292	9 (52.9)	8 (47.1)	0.815
Incentives for international students to study in China	10	39 (48.8)	41 (51.2)	0.776	36 (57.1)	27 (42.9)	0.331	5 (45.5)	6 (54.5)	0.361
My parents want me to study in China	18	21 (52.5)	19 (47.5)	0.440	28 (50.0)	28 (50.0)	0.974	31 (53.4)	27 (46.6)	0.602
Some of my friends are studying in China	19	22 (52.4)	20 (47.6)	0.406	25 (55.6)	20 (44.4)	0.326	33 (49.3)	34 (50.7)	0.353
Reasons for not considering to study in Western university										
Cost of education is high	1	47 (51.1)	45 (48.9)	0.397	18 (43.9)	23 (56.1)	0.331	15 (71.4)	6 (28.6)	0.205
Cost of living is high	2	45 (50.0)	45 (50.0)	0.701	23 (51.1)	22 (48.9)	0.247	12 (63.2)	7 (36.8)	0.432
Difficult to get admissions in Western universities	3	33 (50.8)	32 (49.2)	0.325	30 (52.6)	27 (47.4)	0.030	17 (53.1)	15 (46.9)	0.710
Hard to get student visa	4	27 (46.6)	31 (53.4)	0.738	37 (60.7)	24 (39.3)	0.226	16 (45.7)	19 (54.3)	0.832
Worried about my safety and wellbeing	5	26 (44.8)	32 (55.2)	0.766	29 (53.7)	25 (46.3)	0.400	25 (59.5)	17 (40.5)	0.450
Far from my home country	7	15 (62.5)	9 (37.5)	0.482	16 (48.5)	17 (51.5)	0.444	49 (50.5)	48 (49.5)	0.931
I may not integrate with Western cultures	8	15 (83.3)	3 (16.7)	0.738	17 (42.5)	23 (57.5)	0.113	48 (50.0)	48 (50.0)	0.687
I may feel isolated	6	16 (57.1)	12 (42.9)	0.082	14 (48.3)	15 (51.7)	0.252	50 (51.5)	47 (48.5)	0.535

**Table 4:** Career choices and possible influence of gender differences.

	First Choice				Second Choice				Third Choice			
	N = 154 (%)	Male n = 80 (%)	Female n = 74 (%)	*p*-Value	N = 154 (%)	Male n = 80(%)	Female n = 74(%)	*p*-Value	N = 154 (%)	Male n = 80(%)	Female n = 74(%)	*p*-Value
Academia and research	34 (22.1)	24 (30.0)	10 (13.5)	0.148	15 (9.7)	9 (11.3)	6 (8.1)	0.776	12 (7.8)	6 (7.5)	6 (8.1)	0.699
Clinical pharmacy	30 (19.5)	11 (13.8)	19 (25.7)	0.497	31 (20.1)	20 (25.0)	11 (14.9)	0.502	5 (3.2)	3 (3.8)	2 (2.7)	1.00
Community pharmacy	7 (4.5)	2 (2.5)	5 (6.8)	0.857	14 (9.1)	6 (7.5)	8 (10.8)	0.852	16 (10.4)	7 (8.8)	9 (12.2)	0.299
Drug quality control	10 (6.5)	8 (10.0)	2 (2.7)	0.711	13 (8.4)	8 (10.0)	5 (6.8)	0.724	16 (10.4)	9 (11.3)	7 (9.5)	0.470
Drug regulatory bodies					10 (6.5)	7 (8.8)	3 (4.1)	1.00	11 (7.1)	6 (7.5)	5 (6.8)	0.177
Hospital pharmacy	12 (7.8)	5 (6.3)	7 (9.5)	0.268	18 (11.7)	6 (7.5)	12 (16.2)	0.820	15 (9.7)	6 (7.5)	9 (12.2)	0.456
Medical representative	3 (1.9)	2 (2.5)	1 (1.4)	0.667	1 (0.6)	1 (1.3)			8 (5.2)	4 (5.0)	4 (5.4)	0.886
Pharmaceutical industry	40 (26.0)	22 (27.5)	18 (24.3)	0.946	29 (18.8)	12 (15.0)	17 (23.0)	0.180	22 (14.3)	13 (16.3)	9 (12.2)	0.393
Public health	4 (2.6)	2 (2.5)	2 (2.7)	0.333	11 (7.1)	5 (6.3)	6 (8.1)	0.931	16 (10.4)	7 (8.8)	9 (12.2)	0.470
Working outside home country	11 (7.1)	3 (3.8)	8 (10.8)	0.630	10 (6.5)	5 (6.3)	5 (6.8)	0.690	26 (16.9)	16 (20.0)	10 (13.5)	0.698
Not working	1 (0.6)	0	1 (1.4)	-	1 (0.6)	0	1 (1.4)		1 (0.6)	1 (1.3)	0	-
Others	2 (1.3)	1 (1.3)	1 (1.4)	1.00	1 (0.6)	1 (1.3)	0		6 (3.9)	2 (2.5)	4 (5.4)	1.00

**Table 5:** Influence of age on career choices.

	First Choice					Second Choice					Third Choice				
	N = 154 (%)	≤20 N = (%)	21–30 N = (%)	>30 N = (%)	*p*-Value	N = 154 (%)	≤20	21–30	>30	*p*-Value	N = 154 (%)	≤20	21–30	>30	*p*-Value
Academia and research	34 (22.1)	2 (9.5)	20 (17.1)	12 (75.0)	0.388	15 (9.7)	3 (14.3)	11 (9.4)	1 (6.3)	0.683	12 (7.8)	1 (4.8)	11 (9.4)	1 (6.3)	0.469
Clinical pharmacy	30 (19.5)	4 (19.0)	24 (20.5)	2 (12.5)	0.988	31 (20.1)	4 (19.0)	24 (20.5)	3 (18.8)	0.751	5 (3.2)	0	5 (4.3)	0	0.588
Community pharmacy	7 (4.5)	0	7 (6.0)			14 (9.1)	2 (9.5)	11 (9.4)	1 (6.3)	0.409	16 (10.4)	0	15 (12.8)	0	
Drug quality control	10 (6.5)	1 (4.8)	8 (6.8)	1 (6.3)	0.229	13 (8.4)	1 (4.8)	9 (7.7)	3 (18.8)	0.088	16 (10.4)	2 (9.5)	13 (11.1)	1 (6.3)	0.333
Drug regulatory bodies	0	0	0	0		10 (6.5)	2 (9.5)	6 (5.1)	2 (12.5)	0.113	11 (7.1)	1 (4.8)	6 (5.1)	4 (25.0)	0.337
Hospital pharmacy	12 (7.8)	3 (14.3)	9 (7.7)	0	0.926	18 (11.7)	3 (14.3)	15 (12.8)	0	0.515	15 (9.7)	3 (14.3)	11 (9.4)	1 (6.3)	0.147
Medical representative	3 (1.9)		3 (2.6)	0		1 (0.6)		1 (0.9)	0		8 (5.2)	3 (14.3)	5 (4.3)	0	0.101
Pharmaceutical industry	40 (26.0)	6 (28.6)	33 (28.2)	1 (6.3)	0.367	29 (18.8)	4 (19.0)	21 (17.9)	4 (25.0)	0.259	22 (14.3)	3 (14.3)	15 (12.8)	4 (25.0)	0.780
Public health	4 (2.6)		4 (3.4)	0		11 (7.1)	1 (4.8)	8 (6.8)	2 (12.5)	0.350	16 (10.4)	3 (14.3)	12 (10.3)	1 (6.3)	0.855
Working outside home country	11 (7.1)	4 (19.0)	7 (6.0)	0	0.345	10 (6.5)	1 (4.8)	9 (7.7)	0	0.117	26 (16.9)	4 (19.0)	18 (15.4)	4 (25.0)	0.916
Not working	1 (0.6)	0	1 (0.9)	0		1 (0.6)	0	1 (0.9)	0	-	1 (0.6)	0	1 (0.9)	0	
Others	2 (1.3)	1 (4.8)	1 (0.9)	0	0.317	1 (0.6)	0	1 (0.9)	0	-	6 (3.9)	1 (4.8)	5 (4.3)	0	0.143

**Table 6:** Influence of level of study of respondents on choice of future career.

	First Choice					Second Choice					Third Choice				
	N = 154 (%)	UG N = 109 (%)	M N = 29 (%)	D N = 16 (%)	*p*-Value	N = 154 (%)	UG N = 109 (%)	M N = 29 (%)	D N = 16 (%)	*p*-Value	N = 154 (%)	UG N = 109 (%)	M N = 29 (%)	D N = 16 (%)	*p*-Value
Academia and research	34 (22.1)	9 (8.3)	13 (44.8)	12 (75.0)	0.795	15 (9.7)	11 (10.1)	3 (10.3)	1 (6.3)	0.429	12 (7.8)	7 (6.4)	4 (13.8)	1 (6.3)	0.360
Clinical pharmacy	30 (19.5)	26 (23.9)	3 (10.3)	1 (6.3)	0.315	31 (20.1)	20 (18.3)	8 (27.6)	3 (18.8)	0.038	5 (3.2)	3 (2.8)	1 (3.4)	1 (6.3)	0.344
Community pharmacy	7 (4.5)	7 (6.4)	0	0		14 (9.1)	12 (11.0)	2 (6.9)	0	0.465	16 (10.4)	14 (12.8)	2 (6.9)	0	1.00
Drug quality control	10 (6.5)	5 (4.6)	5 (17.2)	0	0.047	13 (8.4)	7 (6.4)	3 (10.3)	3 (18.8)	0.124	16 (10.4)	11 (10.1)	5 (17.2)	0	0.777
Drug regulatory bodies		0	0	0		10 (6.5)	7 (6.4)	1 (3.4)	2 (12.5)	0.083	11 (7.1)	4 (3.7)	2 (6.9)	5 (31.3)	0.373
Hospital pharmacy	12 (7.8)	12 (11.0)	0	3 (18.8)		18 (11.7)	16 (14.7)	2 (6.9)	0	0.888	15 (9.7)	13 (11.9)	2 (6.9)	0	0.396
Medical representative	3 (1.9)	1 (0.9)	2 (6.9)	0	0.221	1 (0.6)	0	0	1 (6.3)		8 (5.2)	8 (7.3)	0	0	
Pharmaceutical industry	40 (26.0)	32 (29.4)	5 (17.2)	0	0.864	29 (18.8)	19 (17.4)	6 (20.7)	4 (25.0)	0.583	22 (14.3)	11 (10.1)	7 (24.1)	4 (25.0)	0.913
Public health	4 (2.6)	4 (3.7)	0	0		11 (7.1)	6 (5.5)	4 (13.3)	1 (6.3)	0.906	16 (10.4)	12 (11.0)	3 (10.3)	1 (6.3)	0.230
Work outside home country	11 (7.1)	10 (9.2)	1 (3.4)	0	0.752	10 (6.5)	9 (8.3)	0	1 (6.3)	0.862	26 (16.9)	20 (18.3)	2 (6.9)	4 (25.0)	0.691
Not working	1 (0.6)	1 (0.9)	0	0		1 (0.6)	1 (0.9)	0	0		1 (0.6)	1 (0.9)	0	0	
Others	2 (1.3)	2 (1.8)	0	0		1 (0.6)	1 (0.9)	0	0		6 (3.9)	5 (4.6)	1 (3.4)	0	0.380

UG, undergraduate; M, master’s; D, doctoral (PhD).

## Data Availability

The raw data of the findings presented in this current study can be obtained from the corresponding author upon request.

## Supplementary Materials

The following supporting information can be downloaded at: https://www.mdpi.com/article/10.3390/pharmacy10060149/s1, Questionnaire used; QR code for questionnaire administered to respondents on WeChat.
