# Supplementary material for: Study Destination Choice and Career Preferences of International Students at China Pharmaceutical University
Source: Pharmacy (Basel). 2022 Nov 10;10(6):149. doi: 10.3390/pharmacy10060149 (PMC9680234; doi:10.3390/pharmacy10060149)
Supplement: Supplementary file 1 [file pharmacy-10-00149-s001.zip › pharmacy-1959327-supplementary.pdf]

SUPPLEMENTARY MATERIAL

Study Destination Choice and Career Preferences of International Students in China Pharmaceutical University

Raphael N. Alolga[1*], Said Abasse Kassim[2], and Pierre Dramou[3*]

[1] State Key Laboratory of Natural Medicines, School of Traditional Chinese Pharmacy, Department of Pharmcognosy, China Pharmaceutical University, Nanjing 210009, China

[2] Département de management, Faculté des sciences de l'administration, Université Laval, Québec, QC, Canada

[3] Department of Analytical Chemistry, School of Science, China Pharmaceutical University, Nanjing 210009, China

* Correspondence: alolgara@cpu.edu.cn (RNA), pierred@cpu.edu.cn (PD); Tel.: 13776550643 (RNA), 13776662410 (PD).

QUESTIONNAIRE

Factors that underpin Choice of Pharmacy Education in China and Career Path of Foreign Students

Objective: This questionnaire seeks your input on the above topic. The questionnaire is so designed to capture all information required for the actualization of this topic. Names and other personal details of all participants are not required. **We respect the privacy of all participants and would therefore accordingly protect same. Completion and submission of the completed questionnaire is therefore deemed expression of your consent to participate in this study**. If there are any questions or comments concerning this topic, please contact Raphael N. Alolga, PhD (Dept of Pharmacognosy, CPU, email: alolgara@cpu.edu.cn).

Section A: Socio-demographic characteristics

**Gender**	**Participant response**
Male	
Female	
Neutral	
**Age (year)**	
<= 20	
21-30	
31-40	
Above 41	
**Level of study**	
Doctorate	
Master	
Undergraduate	
**Sources of funding**	
Self-funded	
Scholarship from home country government/company	
Scholarship from Chinese government/university	

Section B: Factors influencing choice to study in China.

Rank the following in order of significance based on the Scale*: 1= most important; 2= somewhat important; 3= not important*

**Factor**	**Participant response**		
	**1**	**2**	**3**
The quality of university learning environment			
The quality of academic staff			
Full or partial scholarship from Chinese government			
Wanting to obtain international qualification			
Being able to learn Mandarin language			
Cost of living (e.g., accommodation, food)			
Personal safety and wellbeing			
Ranking of the university			
The growth of Chinese economy			
Learning Asian culture			

Trade agreements between my country and China			
Employment prospects in Asia or beyond			
Ease of entry			
Easy to get study visa			
Being able to start business with Chinese counterpart			
Low cost of the degree			
Incentives for international students to study in China			
My parents want me to study in China			
Some of my friends are studying in China			

Section C: Reasons for not considering to study in Western university.

Rank the following in order of importance according to the Scale*: 1= most important; 2= somewhat important; 3= not important.*

**Factor**	**Participant response**		
	**1**	**2**	**3**
Cost of degree is high			
Cost of living is high			
Difficult to get admissions in Western universities			
Hard to get student visa			
Worried about my safety and wellbeing			
Far from my home country			
I may not integrate with Western cultures			
I may feel isolated			

Section D: Career domain preferred by students

Choose your best 3 options and Rank them in order of importance*: 1= first choice; 2=second choice; 3= third choice.*

**What is your choice career domain after graduation?**	**Rank your response**
Academia and research	
Clinical pharmacy	
Community pharmacy	
Drug quality control	
Drug regulatory bodies	
Hospital pharmacy	
Medical representative	
Pharmaceutical Industry	
Public health	
Working outside of home country	
Not working	
Others:	

Section E: Factors influencing future career domain choice

Rank the following factors in order of importance according to the Scale: *1= Strongly agree; 2= Agree; 3=Neutral; 4=Disagree; 5= Strongly disagree.*

**Factor**	**Participant response**				
	**1**	**2**	**3**	**4**	**5**
**Faculty related influences**					
1. Curriculum course/subject					
2. Faculty extracurricular activities					
3. Faculty member advise					
4.Visit to a workplace (Pharmacy, industry, etc.)					
5. Training in a work place (Pharmacy, industry, etc.)					
**Personal related influences**					
6. Family member/ relatives advise					
7. A family member career choice					
8.A friend's career choice					
9. Good social status					
10. Interaction with practicing pharmacist					
**Job related influences**					
11. Opportunity for self-employment					
12. Opportunity for part time work					
13. Opportunity for promotion and advancement					
14. Opportunity for Health insurance					
15. Job salary and incentives					
16. Job allowances (Car, House)					

QR code of Questionnaire that was scanned by all participants using WeChat
